# MicroRNA-34a Mediates High-Fat-Induced Hepatic Insulin Resistance by Targeting ENO3

**DOI:** 10.3390/nu15214616

**Published:** 2023-10-31

**Authors:** Yuanyuan Wang, Xue Zhao, Liuchao Zhang, Chunxiao Yang, Kening Zhang, Zhuo Gu, Haiyan Ding, Shuangshuang Li, Jian Qin, Xia Chu

**Affiliations:** 1 Department of Nutrition and Food Hygiene, School of Public Health, Key Laboratory of Precision Nutrition and Health, Ministry of Education, Harbin Medical University, Harbin 150081, China; yy950505@hrbmu.edu.cn (Y.W.); 202201064@hrbmu.edu.cn (X.Z.); 2021020237@hrbmu.edu.cn (C.Y.); 2021020232@hrbmu.edu.cn (K.Z.); 2021020281@hrbmu.edu.cn (Z.G.); 2022020149@hrbmu.edu.cn (H.D.); 2022020237@hrbmu.edu.cn (S.L.); 2022020159@hrbmu.edu.cn (J.Q.); 2 Department of Epidemiology and Biostatistics, School of Public Health, Harbin Medical University, Harbin 150081, China; zhangliu@hrbmu.edu.cn

**Keywords:** miR-34a, hepatic insulin resistance, high fat, ENO3

## Abstract

The etiology of numerous metabolic disorders is characterized by hepatic insulin resistance (IR). Uncertainty surrounds miR-34a’s contribution to high-fat-induced hepatic IR and its probable mechanism. The role and mechanism of miR-34a and its target gene ENO3 in high-fat-induced hepatic IR were explored by overexpressing/suppressing miR-34a and ENO3 levels in in vivo and in vitro experiments. Moreover, as a human hepatic IR model, the miR-34a/ENO3 pathway was validated in patients with non-alcoholic fatty liver disease (NAFLD). The overexpression of hepatic miR-34a lowered insulin signaling and altered glucose metabolism in hepatocytes. In contrast, reducing miR-34a expression significantly reversed hepatic IR indices induced by palmitic acid (PA)/HFD. ENO3 was identified as a direct target gene of miR-34a. Overexpression of ENO3 effectively inhibited high-fat-induced hepatic IR-related indices both in vitro and in vivo. Moreover, the expression patterns of members of the miR-34a/ENO3 pathway in the liver tissues of NAFLD patients was in line with the findings of both cellular and animal studies. A high-fat-induced increase in hepatic miR-34a levels attenuates insulin signaling and impairs glucose metabolism by suppressing the expression of its target gene ENO3, ultimately leading to hepatic IR. The miR-34a/ENO3 pathway may be a potential therapeutic target for hepatic IR and related metabolic diseases.

## 1. Introduction

Insulin resistance (IR) refers to the decrease in the efficiency of insulin to promote glucose uptake and utilization for various reasons. The liver plays a central role in the coordination of systemic metabolic homeostasis. When IR occurs in the liver, the body experiences substantial disturbances in glycolipid metabolism, and thus, hepatic IR is closely associated with many of the metabolic diseases currently plaguing our society, such as nonalcoholic fatty liver disease (NAFLD) and type 2 diabetes (T2D) [1,2]. Hepatic IR may precede any change in systemic IR, inflammation or adipose tissue mass [3]. Therefore, clarifying the pathogenesis of hepatic IR will contribute to the early prevention and treatment of multiple metabolic diseases.

MicroRNAs (miRNAs) are a large family of small non-coding RNAs that are considered to be post-transcriptional regulators of gene expression, and they control many developmental and cellular processes in eukaryotes [4]. MiRNA binds to mRNA through base pairing and acts by inhibiting the translation of mRNAs from target genes or by promoting their deadenylation and subsequent degradation [5]. Many miRNAs have been reported to be involved in IR [6]. For instance, Song et al. found that the downregulation of miR-592 in the liver of obese mice and humans contributed to IR by targeting the mRNA of the hepatic FOXO1 [7]. Du et al. reported that miRNA-125b impaired hepatocyte insulin sensitivity by targeting the 3’-untranslated region (3’-UTR) of the mRNA for PIK3CD [8]. Xu et al. showed that a high level of miR-103 indirectly results in hypertriglyceridemia due to IR by increasing adipogenesis in hepatocytes, thereby promoting the development of NAFLD [9]. MiR-34a, one of the most concerning miRNAs, has been reported to be dysregulated in a variety of cancers and plays crucial roles in suppressing tumorigenesis and tumor progression by regulating different target genes [10]. In addition to its key functions in cancer, miR-34a has also been implicated in other noncancerous diseases, such as some metabolic diseases [11]. Some studies have reported that the circulating level of miR-34a is significantly elevated in patients with T2D, and it is positively correlated with homeostatic model assessment (HOMA-IR) [12,13,14]. However, the roles and mechanisms of miR-34a in T2D remain unclear. Our previous study also showed that miR-34a levels were significantly increased in the liver tissues of high-fat diet (HFD)-induced hepatic IR mice [15]. Therefore, we speculate that miR-34a may play an important role in hepatic IR.

In this study, we focused on the roles and mechanisms of miR-34a and its target gene in high-fat-induced hepatic IR based on human, animal and in vitro evidence. Our results suggested that miR-34a is involved in high-fat-induced hepatic IR by affecting the expression of its target gene ENO3. These findings suggest that miR-34a/ENO3 may be a potential novel therapeutic target for hepatic IR-related metabolic diseases, which provides a new perspective on the pathogenesis of hepatic IR.

## 2. Materials and Methods

### 2.1. Human Liver and Serum Samples

The human liver and serum tissues were obtained from 5 patients pathologically diagnosed as NAFLD and 5 control subjects, who underwent hepatic hemangioma surgery at the Affiliated Tumor Hospital of Harbin Medical University (Harbin, China). The liver and serum samples from patients with alcoholic hepatitis, viral hepatitis, severe fibrosis or cirrhosis were excluded. This study was approved by the Ethics Committee of the Cancer Hospital Affiliated to Harbin Medical University (Approval No. 2019-09-R). The purpose and procedure of the study were explained to the subjects prior to sample collection. Written informed consent was obtained from each participant, and the study protocol complied with the ethical norms of the 1975 Declaration of Helsinki.

### 2.2. Animal Experiments

Male 8-week-old C57BL/6J mice (Vital River Laboratory Animal Technology, Beijing, China) were placed in a pathogen-free isolation facility at 22 Tech °C and were free to drink water during a 12 h light/dark cycle. To explore the role of miR-34a in HFD-induced hepatic IR in vivo, miR-34a mimic and inhibitor delivered by a recombinant adeno-associated virus serotype 8 (AAV8) vector containing a liver-specific albumin promoter (Hanbio, Shanghai, China) were used in this study. After 1 week of adaptive feeding, the mice were randomly divided into four groups: normal diet (ND), ND+miR-34a, HFD and HFD + miR-34a inhibitor (*n* = 8 per group). At 12 weeks of feeding with ND or HFD, AAV8-mediated miR-34a mimic or inhibitor was administered to ND and HFD mice by a single injection into the tail vein (1 × 10^12^ v.g./mL in a total volume of 100 μL/mouse), respectively. A scrambled sequence was used as a negative control. Then, the mice continued to be fed ND or HFD for 6 weeks. The diet formulas were shown in Supplementary Table S1. Moreover, the AAV8-mediated liver-specific ENO3 overexpression vector (Hanbio, Shanghai, China) was also used to assess the role of ENO3 in hepatic IR induced by HFD in mice. The mice were randomly divided into four groups: ND, ND+ENO3, HFD and HFD+ENO3 (*n* = 10 per group). At 11 weeks of feeding with ND or HFD, AAV8 vectors carrying overexpression of ENO3 or scrambled sequence were injected in ND and HFD mice by a single injection into the tail vein (1 × 10^12^ v.g./mL in a total volume of 100 μL/mouse), respectively. Then, the mice continued to be fed ND or HFD for 8 weeks. At the end of the experiment, all mice were subjected to an intraperitoneal glucose tolerance test (IPGTT). After sacrifice, blood samples and liver tissue were quickly collected for analysis. All animals received humane care according to the criteria outlined in the Guide for the Care and Use of Laboratory Animals, and the experimental protocol was approved by the Ethics Committee of Harbin Medical University.

### 2.3. IPGTT

IPGTTs were performed as described previously [13]. In brief, the mice were fasted overnight and then given glucose by intraperitoneal injection (2 g/kg body weight). Blood samples were obtained at 0, 30, 60, 90 and 120 min, and serum glucose was measured with an Accu-Chek Performa glucometer (Roche Diagnostics GmbH, Mannheim, Germany). The area under the curve (AUC) of blood glucose between 0 and 120 min was calculated.

### 2.4. Serum Lipids, Insulin and Homeostasis Model Assessment (HOMA)-IR

Serum total cholesterol (TC), triacylglycerol (TG), high-density lipoprotein cholesterol (HDL-C) and low-density lipoprotein cholesterol (LDL-C) were examined with an automatic analyzer (HITACHI-700, Tokyo, Japan) using kits purchased from Biosino Biotechnology Co. (Beijing, China). Mouse insulin was measured with a rat/mouse insulin ELISA kit (LINCO Research, St Charles, MO, USA). HOMA-IR = fasting glucose (mmol/L) × fasting insulin (mU/L)/22.5.

### 2.5. Hematoxylin–Eosin (HE) and Immunohistochemical (IHC) Staining

HE and IHC staining were conducted according to routine procedures. For HE staining, in brief, after deparaffinization and rehydration, 5 μm longitudinal sections were stained with hematoxylin solution for 5 min followed by 5 dips in 1% acid ethanol and then rinsed in distilled water. Then, the sections were stained with eosin solution for 3 min and followed by dehydration with graded alcohol and clearing in xylene [16]. For IHC staining, the treated paraffin sections were incubated at room temperature with 3% H_2_O_2_ for 10 min, washed and sealed at room temperature with 10% BSA for 15 min, incubated with the primary antibody overnight at 4 °C, then added the working solution of secondary antibody and incubated at 37 °C for 30 min followed by 3,3′-diaminobenzidine and hematoxylin staining, respectively. The primary antibodies were as follows: p-Akt (Ser473) (1:100, Cell Signaling Technology, Beverly, MA, USA); ENO3 (1:100, Sangon Biotech, Shanghai, China). The tissue sections were photographed and preserved in a digital scanning microscopic imaging system (Precipoint M8, PreciPoint GmbH, Garching bei München, Germany).

### 2.6. Cell Culture, Treatment and Transfection

Alpha mouse liver 12 (AML12) cells (ATCC, Manassas, VA, USA) were cultured in DMEM/F-12 (HyClone, South Logan, UT, USA) supplemented with 10% fetal bovine serum (PAA Laboratories, Pasching, Austria), 40 ng/mL dexamethasone (Sigma, St. Louis, MO, USA), 1 × Insulin-Transferrin-Selenium (ITS; Gibco, Carlsbad, CA, USA), 100 IU/mL penicillin and 100 μg/mL streptomycin (Beyotime, Shanghai, China). The cells were cultured in a 5% CO_2_ incubator at 37 °C.

Palmitic acid (PA), a major saturated fatty acid, was used to construct a model of high-fat-induced hepatic IR in vitro [17]. To determine the role of miR-34a and ENO3 in high-fat-induced hepatic IR in vitro, miR-34a mimic, miR-34a inhibitor (GenePharma, Shanghai, China), ENO3 siRNA and ENO3 overexpression plasmid (RiboBio, Guangzhou, China) were applied in cell experiments. Specifically, the AML12 cells were transfected or co-transfected with 100 nM miR-34a mimic, 200 nM miR-34a inhibitor, 1 μg/mL ENO3 overexpression plasmid or 100 nM three pairs of siRNA targeted to ENO3 gene (siRNA-1, siRNA-2 and siRNA-3; their target sequences are listed in Supplementary Table S2) using Lipofectamine 2000 reagent (Invitrogen, Carlsbad, CA, USA) according to the manufacturer’s instructions for 24 h or 48 h. The scrambled sequence was used as an NC. After transfection, the cells were treated with or without 400 μM PA for 24 h, and then they were used for the subsequent experiments.

### 2.7. Cellular Glucose Uptake, Glucose Consumption, Glucose Production and Glycogen Content Assays

Cellular glucose uptake was measured using the fluorescent glucose analog 2-NBDG (Invitrogen, CA, USA). In brief, after washing with PBS, the AML12 cells with different treatments were incubated in glucose-free medium (Solarbio, Beijing, China) supplemented with 100 nM insulin (Sigma, St. Louis, MO, USA) for 15 min and with 0.1 mM 2-NBDG for 30 min at 37 °C. Then, the cells were stained with Hoechst 33,342 (Beyotime, Shanghai, China) at room temperature for 30 min. After washing with PBS, photos were taken with a confocal laser microscope (A1; Nikon, Tokyo, Japan) and saved. Glucose consumption was calculated by detecting the glucose content in the culture medium before and after the different treatments using a Glucose Assay kit (Solarbio, Beijing, China). For the glucose production test, after different treatments, the AML12 cells were incubated in a glucose-free medium for 4 h, and then the glucose content in the culture medium was detected by Glucose Assay kit (Solarbio, Beijing, China). Cellular glycogen content was determined using the Glycogen Content Assay kit (Solarbio, Beijing, China) according to the manufacturer’s protocol. All experiments were performed at least three times, and the data were normalized by the protein content.

### 2.8. Target Prediction and Luciferase Activity Assay

The target genes of miR-34a were predicted with TargetScan 7.2 (http://www.targetscan.org/, accessed on 8 July 2019) [18]. The Dual-Luciferase reporter plasmid (pmiR-RB-REPORT^TM^) carrying the wild-type (WT) or mutated (MUT) 3′ UTR of the ENO3 gene was constructed (GeneCreate, Wuhan, China). HEK-293 cells (ATCC, Manassas, VA, USA) were co-transfected with 200 ng of recombinant plasmid, 100 nM miR-34a mimic and 200 nM miR-34a inhibitor. A scrambled sequence was used as an NC. After 48 h of transfection, the luciferase activities were measured with a Dual-Luciferase reporter assay kit (Promega, Madison, WI, USA) on a luminometer (GloMaxTM 20/20, Promega) [19].

### 2.9. Western Blot

Western blot was performed as described in [20]. The primary antibodies used were as follows: Akt (1:1000), p-Akt (Ser473) (1:2000) and β-actin (1:1000) (Cell Signaling Technology, Beverly, MA, USA); IRS2 (1:1000) and p-IRS2 (Ser1100) (1:1000) (Absin, Shanghai, China); ENO3 (1:2000, Sangon Biotech, Shanghai, China). The secondary antibody was goat anti-rabbit IgG (1:8000, Sangon Biotech, Shanghai, China).

### 2.10. Quantitative Real-Time PCR

Total RNA was isolated from liver tissue and cells with TRIzol reagent (Invitrogen, Carlsbad, CA, USA) according to the manufacturer’s instructions. Total RNA was reverse transcribed to cDNA using a High-Capacity cDNA Reverse Transcription Kit (Applied Biosystems, Foster City, CA, USA). Real-time PCR was performed with the SYBR Green PCR Master Mix using a 7500 FAST Real-time PCR System (Applied Biosystems, Foster City, CA, USA). The expression of U6 or β-actin was used as an internal control. All primers were synthesized by Sangon Biotech (Shanghai, China), and the sequences were shown in Supplemental Table S3.

### 2.11. Statistical Analysis

Values are presented as mean ± SD. All data shown were representative of at least three individual experiments. Statistical analyses were performed with SPSS 25 software (SPSS, Chicago, IL, USA). Significance was determined by using a two-tailed Student *t*-test or one-way ANOVA as appropriate. *p*-values of <0.05 were considered significant.

## 3. Results

### 3.1. Increased miR-34a Mediates PA-Induced IR in AML12 Cells

**Figure 1:**
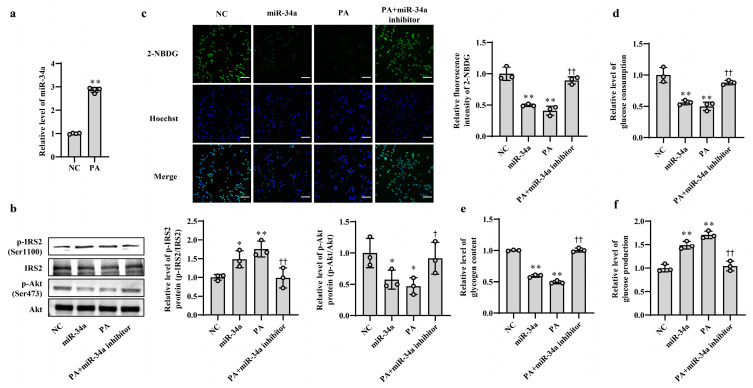
Increased miR-34a mediates PA-induced IR in AML-12 cells. (**a**) The changes in the level of miR-34a in AML-12 cells treated with or without 400 μM PA for 24 h. (**b**–**f**) The changes in the relative protein expression of p-IRS2 (Ser1100) and p-Akt (Ser473) (**b**), glucose uptake (scale bars, 100 μM) (**c**), glucose consumption (**d**), glycogen content (**e**) and glucose production (**f**) in AML-12 cells with different treatments. The AML-12 cells were transfected with 100 nM scrambled sequence, 100 nM miR-34a mimic or 200 nM miR-34a inhibitor for 24 h and then treated with or without 400 μM PA for 24 h. A scrambled sequence was used as a negative control (NC). * *p* < 0.05 and ** *p* < 0.01 vs. NC; † *p* < 0.05 and †† *p* < 0.01 vs. PA.

In our previous study, we found that the expression level of miR-34a was significantly elevated in liver tissues of HFD-induced hepatic IR in mice [15]. In this study, PA, which is one of the main saturated fatty acids, was used to treat AML12 cells for simulating HFD-induced hepatic IR model in vitro. The results showed that the level of miR-34a was markedly increased in AML12 cells treated with PA (Figure 1a), which was consistent with the in vivo results. Next, to clarify the role of miR-34a in PA-induced hepatic IR, miR-34a mimic and miR-34a inhibitor were used in AML12 cells with or without PA treatment to increase or inhibit the expression level of miR-34a. Compared with NC group, cells transfected with the miR-34a mimic demonstrated a significant promotion of miR-34a expression (Supplementary Figure S1). The relative expression levels of p-IRS2 (Ser1100) and p-Akt (Ser473) proteins were upregulated and downregulated, respectively (Figure 1b), and cellular glucose uptake was correspondingly reduced (Figure 1c). Moreover, cellular glucose consumption and intracellular glycogen content were decreased, and glucose production was increased in AML12 cells transfected with miR-34a mimic (Figure 1d–f). As expected, IR occurred in AML12 cells treated with PA, reflected by enhancements in p-IRS2 (Ser1100) protein expression and glucose production and reductions in the p-Akt (Ser473) protein level, glucose uptake, glucose consumption and intracellular glycogen content. These results are similar to those of the miR-34a mimic transfection group. However, cotreatment with the miR-34a inhibitor eliminated these changes triggered by PA (Figure 1b–f). These results suggest that miR-34a is involved in PA-induced hepatocyte IR in AML12 cells.

### 3.2. MiR-34a Contributes to HFD-Induced Hepatic IR in Mice

**Figure 2:**
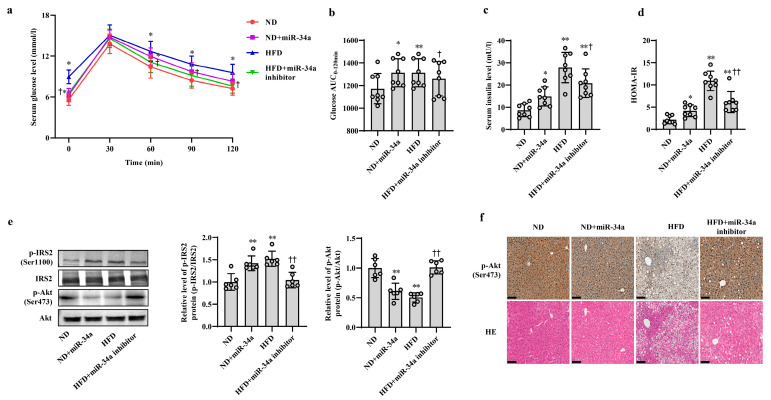
MiR-34a contributes to HFD-induced hepatic IR in mice. (**a**–**f**) The changes in blood glucose during IPGTT (**a**), the AUC of glucose between 0 and 120 min of IPGTT (**b**), the levels of serum insulin (**c**), HOMA-IR values (**d**), the expression level of p-IRS2 (Ser1100) and p-Akt (Ser473) protein detected by Western blot (**e**), the expression level of p-Akt (Ser473) protein detected by immunohistochemical staining (scale bars, 100 μM) and HE staining of liver tissue (scale bars, 100 μM) (**f**) in the liver of mice of different groups. After 12 weeks of feeding with ND or HFD, the mice were administered AAV8-mediated liver-specific miR-34a mimic or inhibitor by tail vein, respectively. Then, the mice continued to be fed an ND or HFD for 6 weeks. * *p* < 0.05 and ** *p* < 0.01 vs. ND; † *p* < 0.05 and †† *p* < 0.01 vs. HFD.

Next, animal experiments were used to validate the effects of miR-34a on HFD-induced hepatic IR in vivo. After 12 weeks of ND and HFD feeding, the mice were injected with AAV8-mediated liver-specific miR-34a mimic or inhibitor via the tail vein, and then fed their corresponding diets for another 6 weeks. Compared with those in the ND group, the body weight and serum levels of TG, TC, HDL-C and LDL-C in the HFD group were significantly increased; however, neither the miR-34a mimic nor miR-34a inhibitor interventions affected the changes in these indicators (Supplemental Table S4, Supplementary Figure S2). At the end of the experiment, all mice were subjected to IPGTT. Except for those at 30 min, the levels of blood glucose in four groups at other time points (0, 60, 90 and 120 min) showed different degrees of changes (Figure 2a). Subsequently, the AUC values of glucose were calculated from 0 and 120 min. The results showed that the AUC values were significantly higher in the ND + miR-34a and HFD groups than in the ND group, while the AUC values in the HFD + miR-34a inhibitor group were statistically lower than those in the HFD group (Figure 2b). Furthermore, the serum insulin levels and HOMA-IR values were significantly increased to varying degrees in the miR-34a overexpression and HFD groups compared with those in the ND group, but the intervention of miR-34a inhibitor markedly suppressed the increases in these indices in the HFD group (Figure 2c,d). Moreover, the changes in p-IRS2 (Ser1100) and p-Akt (Ser473) protein expression in liver tissues were also similar to the results of cellular experiments. Specifically, the injection of miR-34a mimic and HFD increased p-IRS2 (Ser1100) protein level and suppressed p-Akt (Ser473) protein expression in mouse liver; however, the miR-34a inhibitor effectively reduced the HFD-induced increase in p-IRS2 (Ser1100) protein levels and decrease in p-Akt (Ser473) protein levels (Figure 2e,f). In addition, liver histopathological analyses showed swollen hepatocytes and a large number of spherical lipid droplets in the cytoplasm of HFD-fed mice compared with ND mice, while suppression of miR-34a by the miR-34a inhibitor markedly improved these changes (Figure 2f). The above results reveal that miR-34a is involved in the hepatic IR induced by HFD feeding in mice.

### 3.3. ENO3 Expression Is Specifically Reduced by miR-34a as a Direct Target Gene

**Figure 3:**
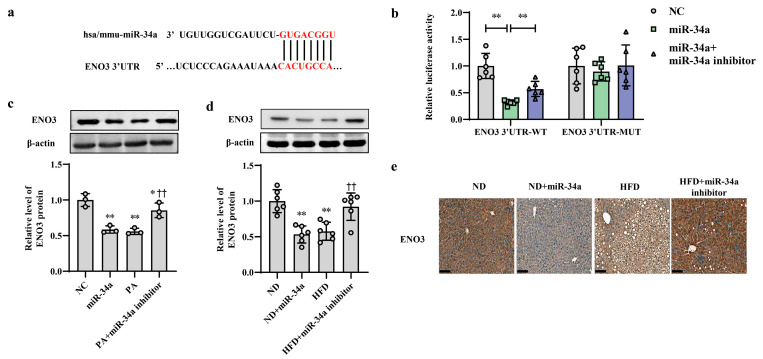
ENO3 is specifically repressed by miR-34a as a direct target gene. (**a**) Sequence alignment between miR-34a and the 3′UTR of ENO3. (**b**) The effect of miR-34a on the 3′UTR of ENO3 was determined by luciferase activity assays. HEK-293 cells were co-transfected with constructed WT or MUT 3′UTR of ENO3 plasmid, miR-34a and its inhibitor for 48 h, and luciferase activities were measured. (**c**) The changes in the expression level of ENO3 protein in AML-12 cells with different treatments. The AML-12 cells were transfected with 100 nM miR-34a mimic or 200 nM miR-34a inhibitor for 24 h and then treated with or without 400 μM PA for 24 h. A scrambled sequence was used as a negative control (NC). (**d**,**e**) The changes in the expression level of ENO3 protein detected by Western blot (**d**) and immunohistochemical staining (scale bars, 100 μM) (**e**) in the liver of mice of different groups. After 12 weeks of feeding with ND or HFD, the mice were administered AAV8-mediated liver-specific miR-34a mimic or inhibitor by tail vein, respectively. Then, the mice continued to be fed an ND or HFD for 6 weeks. * *p* < 0.05 and ** *p* < 0.01 for the indicated comparison, or vs. NC or ND; †† *p* < 0.01 vs. PA or HFD.

ENO3 was predicted as a potential candidate target gene of miR-34a by the TargetScan online database (Figure 3a). Moreover, the results from proteomic and Western blot analyses showed that compared with that in the ND group, the hepatic protein level of ENO3 in the HFD group was significantly reduced (Supplementary Figure S3). In addition, in AML-12 cells, the transfection of miR-34a markedly reduced the mRNA and protein expression levels of ENO3 (Supplementary Figure S4). These results suggest that ENO3 may be directly regulated by miR-34a. Next, a luciferase assay was used to analyze the relationship between ENO3 and miR-34a. The results showed that miR-34a overexpression significantly inhibited luciferase activity in HEK-293 cells transfected with a reporter plasmid carrying the WT 3′UTR of the ENO3 gene, while co-transfection of the miR-34a inhibitor reversed the decline in luciferase activity caused by miR-34a overexpression to some extent. The luciferase activity in the ENO3 MUT 3′UTR group was not influenced by miR-34a or co-transfection of miR-34a and the miR-34a inhibitor (Figure 3b). In addition, the regulatory effect of miR-34a on ENO3 was also demonstrated in both in vitro and in vivo models of high-fat-induced hepatic IR. The hepatic expression of ENO3 protein was markedly downregulated in the miR-34a, PA and HFD groups, similar to the results described above. However, the miR-34a inhibitor significantly mitigated the PA/HFD-induced decrease in ENO3 protein expression (Figure 3c–e). These results indicate that ENO3 is a direct target gene of miR-34a.

### 3.4. ENO3 Is Involved in PA- or HFD-Induced Hepatic IR

**Figure 4:**
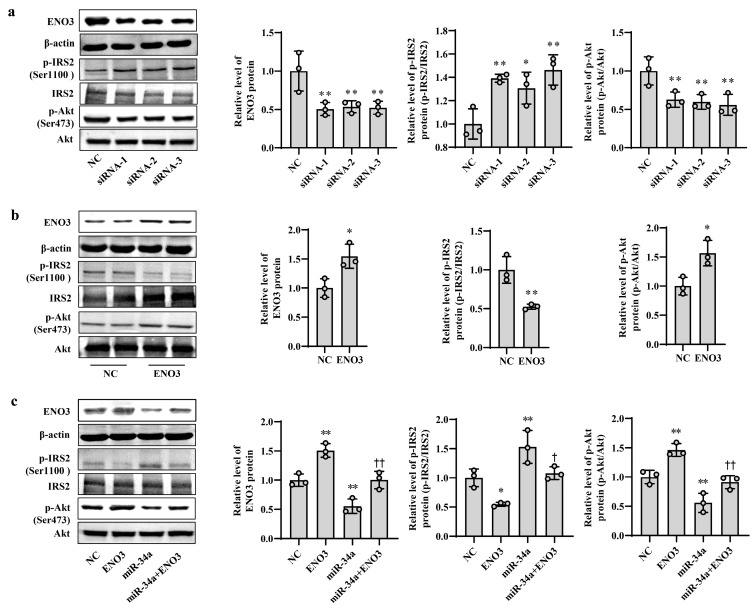
ENO3 participates in miR-34a-mediated hepatic IR in AML12 cells. (**a**) The changes in the expression levels of ENO3, p-IRS2 (Ser1100) and p-Akt (Ser473) proteins in AML-12 cells transfected with ENO3 siRNAs. The AML12 cells were transfected 100 nM three pairs of siRNA targeted to ENO3 gene (siRNA-1, siRNA-2 and siRNA-3, respectively) for 48 h, and related proteins were measured. (**b**) The changes in the expression levels of ENO3, p-IRS2 (Ser1100) and p-Akt (Ser473) proteins in AML12 cells transfected with ENO3 overexpression plasmid. The AML12 cells were transfected 1 μg/mL ENO3 overexpression plasmid for 48 h, and related proteins were detected. (**c**) The changes in the expression levels of ENO3, p-IRS2 (Ser1100) and p-Akt (Ser473) proteins in AML12 cells treated with miR-34a or/and ENO3 overexpression plasmid. The AML12 cells were transfected with 100 nM miR-34a mimic or/and 1 μg/mL ENO3 overexpression plasmid for 48 h, and related proteins were tested. The scrambled sequence was used as a negative control (NC). * *p* < 0.05 and ** *p* < 0.01 vs. NC; † *p* < 0.05 and †† *p* < 0.01 vs. miR-34a.

To clarify the role of ENO3 in hepatic IR, knockdown and overexpression of ENO3 were performed in this study via siRNA interference and plasmid vector transfection, respectively. In AML12 cells, the mRNA and protein expression levels of ENO3 were significantly downregulated in the three groups transfected with ENO3 siRNA. This was accompanied by an increase in p-IRS2 (Ser1100) protein expression and a decrease in p-Akt (Ser473) protein expression, compared with the NC group (Supplementary Figure S5, Figure 4a). Conversely, the mRNA and protein levels of ENO3 were markedly elevated in the ENO3 overexpression plasmid transfection group, and the expression levels of p-IRS2 (Ser1100) and p-Akt (Ser473) proteins were attenuated and enhanced, respectively (Supplementary Figure S6 and Figure 4b). Moreover, our above experiments confirmed that increases in miR-34a promote IR in hepatocytes. We next verified whether ENO3 was able to affect hepatic IR induced by miR-34a. The results showed that with increasing expression of ENO3 protein, both the increase in p-IRS2 (Ser1100) protein levels and the decrease in p-Akt (Ser473) protein levels induced by miR-34a were significantly suppressed (Figure 4c).

**Figure 5:**
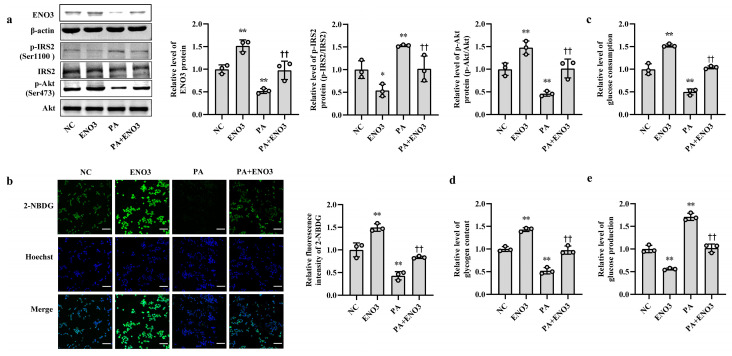
ENO3 is involved in PA-induced hepatic IR in AML12 cells. (**a**–**e**) The changes in the expression levels of ENO3, p-IRS2 (Ser1100) and p-Akt (Ser473) (**a**), glucose uptake (scale bars, 100 μM) (**b**), glucose consumption (**c**), glycogen content (**d**) and glucose production (**e**) in AML-12 cells with different treatments. The AML-12 cells were transfected with 100 nM scrambled sequence, 1 μg/mL ENO3 overexpression plasmid for 24 h and then treated with or without 400 μM PA for 24 h. A scrambled sequence was used as a negative control (NC). * *p* < 0.05 and ** *p* < 0.01 vs. NC; †† *p* < 0.01 vs. PA.

**Figure 6:**
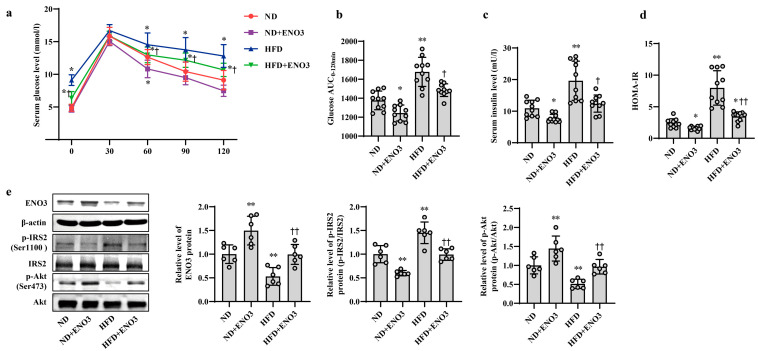
ENO3 is involved in HFD-induced hepatic IR in mice. (**a**–**e**) The changes in blood glucose during IPGTT (**a**), the AUC of glucose between 0 and 120 min of IPGTT (**b**), serum insulin levels (**c**), HOMA-IR values (**d**), the expression levels of ENO3, p-IRS2 (Ser1100), p-Akt (Ser473) proteins (**e**) in the liver of mice of different groups. After 11 weeks of being fed with ND or HFD, the mice were administered AAV8-mediated liver-specific ENO3 expression plasmid or scrambled sequence via the tail vein, respectively. Then, the mice continued to be feed an ND or HFD for 8 weeks. * *p* < 0.05 and ** *p* < 0.01 vs. ND; † *p* < 0.05 and †† *p* < 0.01 vs. HFD.

Furthermore, the effects of ENO3 on high-fat-induced hepatic IR were validated via cell and animal experiments. In AML12 cells, the overexpression of ENO3 evidently ameliorated the changes in IR-related indications triggered by PA, including those in the protein expression levels of p-IRS2 (Ser1100) and p-Akt (Ser473) (Figure 5a) and the levels of cellular glucose uptake, glucose consumption, intracellular glycogen content and glucose production (Figure 5b–e). These effects were similar to those of the miR-34a inhibitor in PA-induced IR. In animal studies, after 11 weeks of ND and HFD feeding, the mice were injected with the AAV8-mediated liver-specific ENO3 overexpression vector via the tail vein and then fed their corresponding diets for another 8 weeks. Compared with those in the ND group, the body weight and serum levels of TG, TC, HDL-C and LDL-C in the HFD group were significantly increased. However, the overexpression of ENO3 reduced the increase in body weight and serum TG levels induced by an HFD (Supplemental Table S5, Supplementary Figure S7). The IPGTT results showed that the levels of serum glucose in the four groups of mice showed varying degrees of changes at different time points (Figure 6a). The intervention of ENO3 overexpression reduced the AUC of glucose during 0–120 min of IPGTT in both the ND and HFD groups (Figure 6b). Moreover, ENO3 overexpression markedly decreased serum insulin levels and HOMA-IR values in ND or HFD-fed mice (Figure 6c,d). In addition, in the liver tissues of ND or HFD-fed mice, increased ENO3 via transfection of the ENO3 overexpression plasmid also decreased and increased the protein expression levels of p-IRS2 (Ser1100) and p-Akt (Ser473), respectively (Figure 6e). Meanwhile, there was no statistically significant effect of ENO3 on miRNA-34a expression with or without high fat (Supplementary Figure S8). The results from in vitro and in vivo studies indicate that ENO3 plays a crucial role in high-fat-induced hepatic IR.

### 3.5. Validation of Changes in miR-34a/ENO3 Pathway in Subjects with Hepatic IR

**Figure 7:**
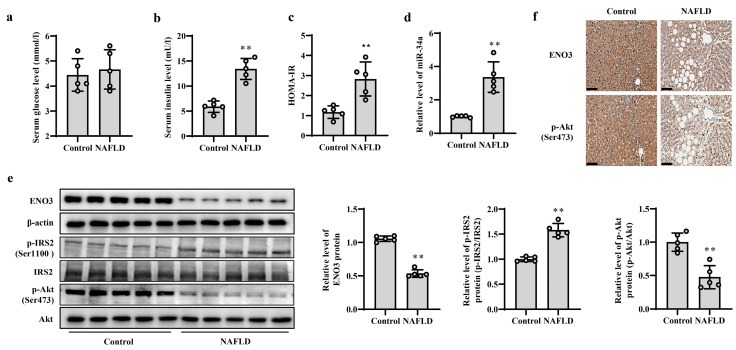
Validation of changes in miR-34a/ENO3 pathway in the liver of patients with NAFLD. The changes in serum levels of glucose (**a**), insulin (**b**), HOMA-IR values (**c**), the level of miR-34a (**d**), the expression levels of ENO3, p-IRS2 (Ser1100) and p-Akt (Ser473) proteins detected by Western blot (**e**) and immunohistochemical staining of ENO3 and p-Akt proteins (scale bars, 100 μM) (**f**) in the liver of control subjects and patients with NAFLD. ** *p* < 0.01 vs. Control.

The occurrence and development of NAFLD is closely related to IR; thus, patients with NAFLD were used as subjects with hepatic IR to verify the changes in the miR-34a/ENO3 pathway in the human body. The basic information of the control subjects and NAFLD patients is given in Supplementary Table S6. The BMI and ALT values in patients with NAFLD were higher than those of control subjects, but there were no significant differences in other indicators between the two groups. Histochemical results show significant steatosis of liver tissue in patients with NAFLD (Supplementary Figure S9). Compared with control subjects, although there was no obvious change in serum glucose levels, serum insulin levels and HOMA-IR values were significantly increased in patients with NAFLD (Figure 7a–c), suggesting the presence of IR in the patients with NAFLD. The results of real-time PCR showed that the expression level of miR-34a in the liver of NAFLD patients was significantly higher than that of control subjects (Figure 7d). Moreover, compared with those in control subjects, the protein expression levels of hepatic ENO3 and p-Akt (Ser473) proteins in patients with NAFLD were significantly reduced, and the protein level of hepatic p-IRS2 (Ser1100) was markedly increased (Figure 7e,f). These results indicate that the change trend of the miR-34a/ENO3 pathway in the body of subjects with hepatic IR is consistent with those obtained in high-fat-induced hepatic IR models in cells and animals.

## 4. Discussion

In this study, we investigated for the first time the role and mechanisms of miR-34a in high-fat-induced hepatic IR. The results showed that high fat intake promoted an increase in miR-34a. This interfered with glucose metabolism by targeting the inhibition of ENO3 expression, which in turn led to the development of hepatic IR. These findings indicate that miR-34a/ENO3 may become a new target for the prevention and treatment of hepatic IR-related diseases.

Studies have shown that long-term application of an HFD can lead to disturbances in glucolipid metabolism and induce IR [21]. In a previous study, we found that miR-34a was significantly increased in liver tissues of mice with HFD-induced hepatic IR [15], and this phenomenon was also confirmed in the study of Elham et al. [22]. In the present study, we applied PA-treated AML12 cells to construct a high-fat-induced hepatic IR cell model and similarly found significantly elevated miR-34a levels. Together with some studies reporting that miR-34a is associated with IR-related diseases, such as T2D and NAFLD [23,24,25]. All of these findings suggest that miR-34a may play an important role in high-fat-induced hepatic IR.

To confirm the role of miR-34a in high-fat-induced hepatic IR, we increased or decreased miR-34a expression in normal and high-fat-induced hepatic IR models in vitro and in vivo, respectively, to assess insulin signaling and glucose metabolism from both positive and negative aspects. IRS/Akt is one of the major signaling pathways in the regulation of metabolic function by insulin and is involved in glucolipid metabolism in a variety of cells. It has been shown that the phosphorylation of serine/threonine residues of IRS inhibits its tyrosine phosphorylation [26,27], impairing downstream insulin signaling, which in turn triggers IR [28]. IRS2 is an important molecular switch that mediates insulin signaling in the liver [29]. The results of both in vitro and in vivo experiments showed that the overexpression of miR-34a significantly increased the expression of p-IRS2 (Ser1100), with a corresponding decrease in the expression levels of downstream p-Akt (Ser473), leading to impaired glucose metabolism. After inhibiting miR-34a in a high-fat-induced hepatic IR model, the expression of p-IRS2 (Ser1100) was significantly downregulated, while its downstream p-Akt (Ser473) expression was significantly increased, leading to impaired glucose metabolism-related indices. These findings suggest that miR-34a is involved in mediating high-fat-induced hepatic IR, and reducing miR-34a may partially alleviate hepatic IR.

MiRNAs perform biological functions by regulating downstream target genes. Therefore, we conducted a series of experiments to screen and validate the downstream target genes regulated by miR-34a. The TargetScan database was used to predict potential target genes of miR-34a. Based on this prediction and the results of protein sequencing from previous experiments on HFD-fed mouse liver tissue, ENO3 was identified as a potential target gene for miR-34a. Subsequent luciferase assays confirmed that ENO3 is a direct target of miR-34a. Both in vivo and in vitro experiments demonstrated that miR-34a-targeted ENO3. During this process, we found that miR-34a not only inhibited the expression of ENO3 protein, but also reduced the level of ENO3 mRNA, suggesting that miR-34a may decrease ENO3 protein expression by degrading its mRNA.

Enolase is a crucial enzyme in the glycolysis process. It catalyzes the transformation of 2-phosphoglycerate to phosphoenolpyruvate in the glycolytic pathway [30,31]. In higher vertebrates, Enolase is a dimeric protein composed of three subunits—α, β and γ—which are encoded by distinct genes. The ENO3 gene encodes β-enolase, which is found in nearly all tissues in the body, but is more abundant in skeletal muscle tissue. Studies have shown that ENO3 may play an important role in muscle development and regeneration, and its deficiency may lead to metabolic myopathies [32]. Theoretically, ENO3 could enhance glucose metabolism and potentially alleviate IR. For instance, Vitor et al. found that the expression of ENO3 was significantly reduced in the skeletal muscle of rats with impaired insulin sensitivity [33]. However, fewer studies have been conducted on the role of ENO3 in hepatic IR, and in particular, there are almost no studies on the involvement of combined miRNAs in mediating it. Therefore, as a direct regulatory target of miR-34a, the role of ENO3 in hepatic IR identified in this study must also be validated. The experimental results confirmed that inhibiting ENO3 expression significantly increased the protein levels of p-IRS2 (Ser1100) and decreased the protein levels of p-Akt (Ser473). Furthermore, overexpressing ENO3 effectively prevented the upregulation of p-IRS2 (Ser1100) protein expression and the decrease in p-Akt (Ser473) protein expression caused by miR-34a/PA/HFD, and improved the glucose metabolism-related indices, thus alleviating the high-fat-induced IR status. All of these results confirm that ENO3 can improve glucose metabolism by enhancing insulin signaling, thereby improving high-fat-induced IR in the liver.

In addition to conducting cellular and animal experiments, we also confirmed the expression of factors in the miR-34a/ENO3 pathway in human liver tissues during IR. Hepatic IR is strongly associated with hepatic lipid accumulation and degeneration [34]. Therefore, we selected liver tissues from NAFLD patients as a model for human hepatic IR. The results showed that compared with those in the control group, the HOMA-IR values were significantly increased and the protein expression levels of p-IRS2 (Ser1100) were increased in the NAFLD group. Additionally, the expression levels of the down-stream p-Akt (Ser473) protein were significantly decreased in liver tissue. These findings suggest that NAFLD patients had impaired insulin signaling in liver tissue and were in a state of IR. In NAFLD patients with hepatic IR, the level of miR-34a was elevated, while the protein expression levels of ENO3 were significantly reduced, which was consistent with the results of animal and cellular experiments. However, there are inconsistencies in the results of recent studies regarding changes in ENO3 expression in NAFLD. For instance, Wen et al. showed an upregulation of ENO3 expression in the liver of patients with NAFLD through bioinformatics analysis of the GEO database [35]. In contrast, Chalasani et al. reported no significant difference in ENO3 expression in liver tissue between individuals with NAFLD and individuals in the normal control group [36]. We speculate that the inconsistencies in the results of the aforementioned studies may be because ENO3 expression is influenced by several factors, such as race and disease progression. Therefore, our future studies will focus on conducting further research and exploring the mechanisms involved in mediating ENO3 in the development of IR in the human liver.

Overall, the findings of this study have led us to propose a new pathway linking high-fat conditions to hepatic IR, namely, the miR-34a/ENO3 pathway. These findings provide a fresh perspective on the progression of high-fat-induced hepatic IR and related metabolic diseases. Therefore, the regulation of miR-34a and its target gene ENO3 may be an effective approach to improve hepatic IR and prevent related metabolic diseases.

## Data Availability

The datasets used and/or analyzed during the current study are available from the corresponding author on reasonable request.

## Supplementary Materials

The following supporting information can be downloaded at: https://www.mdpi.com/article/10.3390/nu15214616/s1, Table S1: The ingredients of normal diet and high-fat diet; Table S2: The siRNA target sequences in this study; Table S3: Primer sequences used for quantitative real-time PCR; Table S4: Basic information on mice treated with or without miR-34a or its inhibitor in each group; Table S5: Basic information on mice treated with or without overexpression of ENO3 in each group; Table S6: Basic information of control subjects and NAFLD patients; Figure S1: The changes in the level of miR-34a in AML-12 cells treated with 100 nM scrambled sequence or 100 nM miR-34a mimic for 48 h; Figure S2: Body weight changes in mice during feeding; Figure S3: The changes in ENO3 protein levels; Figure S4: The changes in the levels of ENO3 mRNA and protein; Figure S5: The changes in the mRNA level of ENO3 in AML-12 cells; Figure S6: The changes in the mRNA level of ENO3 in AML-12 cells treated with 1 μg/mL scrambled sequence or 1 μg/mL ENO3 overexpression plasmid for 48 h; Figure S7: Body weight changes in mice during feeding; Figure S8: ENO3 did not affect miRNA-34a expression; Figure S9: HE staining of liver tissue (scale bars, 100 μM) in the liver of control subjects and patients with NAFLD.
