# Supplementary material for: MicroRNA-34a Mediates High-Fat-Induced Hepatic Insulin Resistance by Targeting ENO3
Source: Nutrients. 2023 Oct 31;15(21):4616. doi: 10.3390/nu15214616 (PMC10650923; doi:10.3390/nu15214616)
Supplement: Supplementary file 1 [file nutrients-15-04616-s001.zip › Supplementary Figure.pdf]

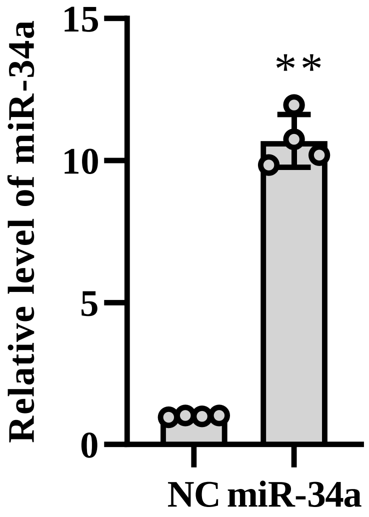

Supplementary Figure 1 The changes in the level of miR-34a in AML-12 cells treated with 100 nM scrambled sequence or 100 nM miR-34a mimic for 48 h. A scrambled sequence was used as a negative control (NC). **$P < 0.01$ vs NC.

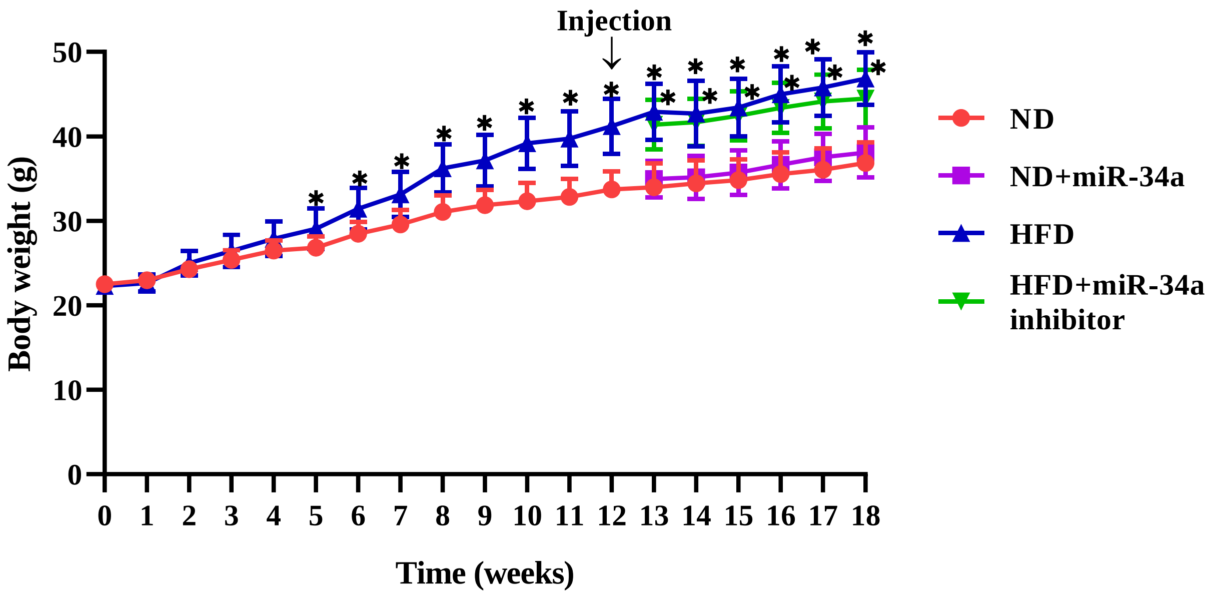

Supplementary Figure 2 Body weight changes in mice during feeding. After 12 weeks of feeding with ND or HFD, the mice were administered AAV8-mediated liver-specific miR-34a mimic or inhibitor by tail vein, respectively. Then, the mice continued to be feed a ND or HFD for 6 weeks. *P < 0.05 vs ND.

a

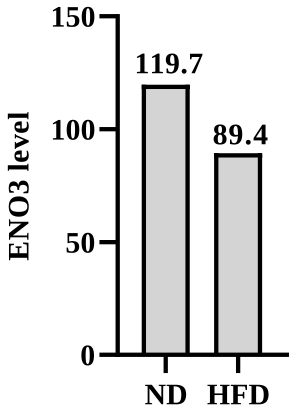

b

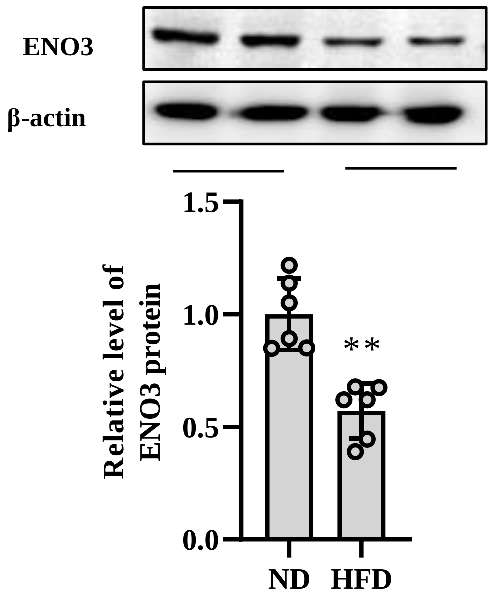

Supplementary Figure 3 The changes in ENO3 protein levels were detected by proteomics (a) and western blot (b). ***P* < 0.01 vs ND.

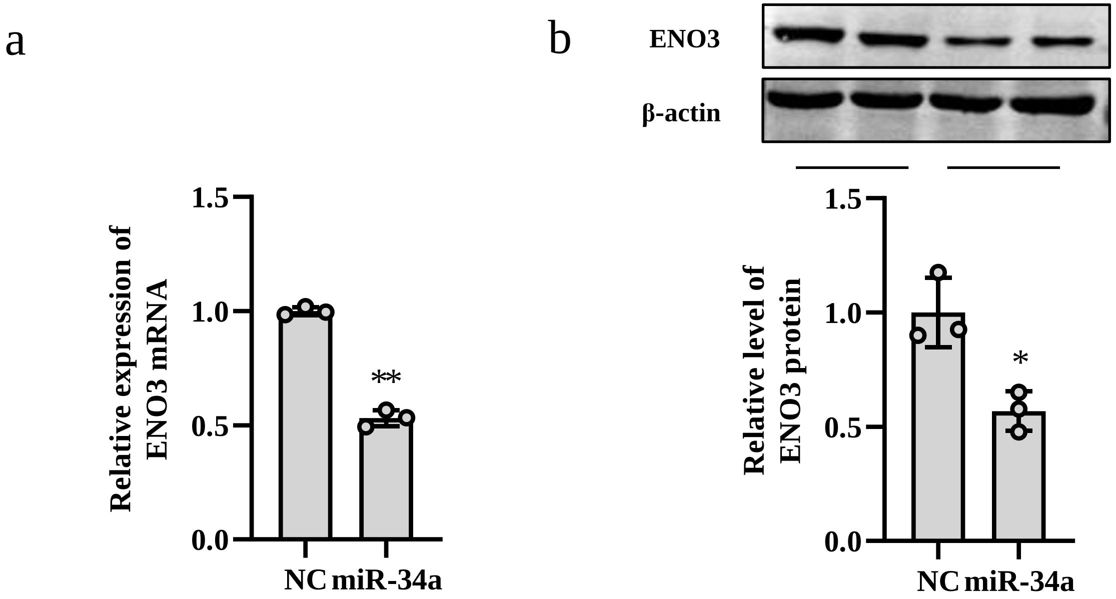

Supplementary Figure 4 The changes in the levels of ENO3 mRNA (a) and protein (b) in the AML-12 cells treated with 100 nM scrambled sequence or 100 nM miR-34a mimic for 48 h. A scrambled sequence was used as a negative control (NC). *P < 0.05 and **P < 0.01 vs NC.

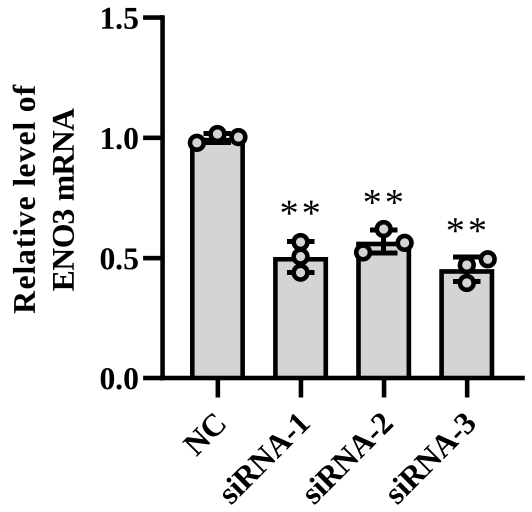

Supplementary Figure 5 The changes in the mRNA level of ENO3 in AML-12 cells treated with 100 nM scrambled sequence or 100 nM three pairs of siRNA targeted to ENO3 gene for 48 h. A scrambled sequence was used as a negative control (NC). **$P < 0.01$ vs NC.

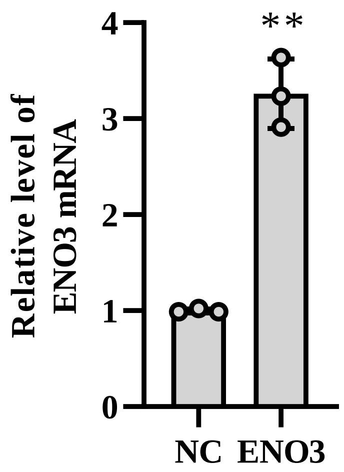

Supplementary Figure 6 The changes in the mRNA level of ENO3 in AML-12 cells treated with 1 μg/mL scrambled sequence or 1 μg/mL ENO3 overexpression plasmid for 48 h. A scrambled sequence was used as a negative control (NC). $^{**}P < 0.01$ vs NC.

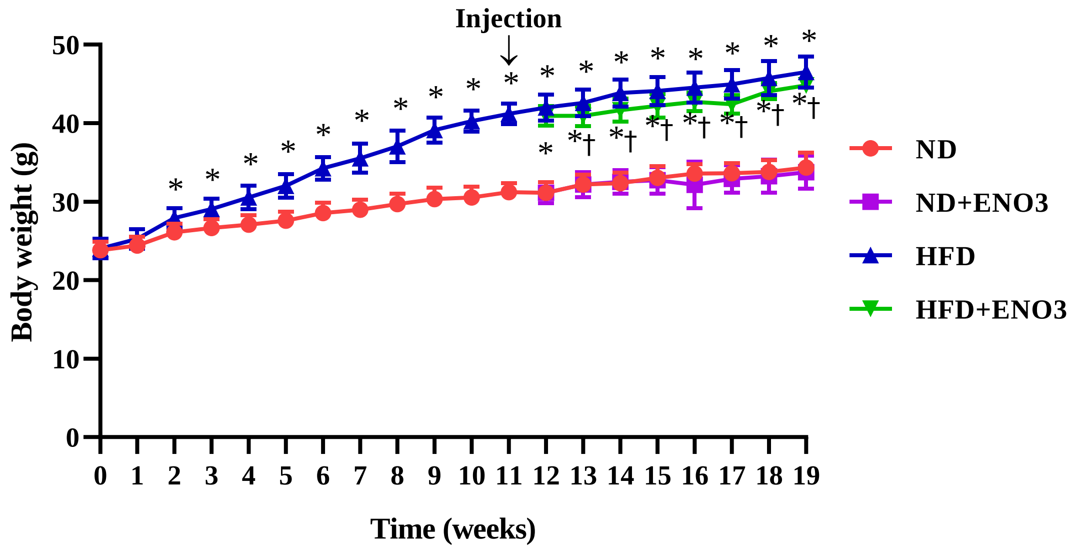

Supplementary Figure 7 Body weight changes in mice during feeding. After 11 weeks of being fed with ND or HFD, the mice were administered AAV8-mediated liver-specific ENO3 expression plasmid or scrambled sequence via the tail vein, respectively. Then, the mice continued to be feed a ND or HFD for 8 weeks. *P < 0.05 vs ND; †P < 0.05 vs HFD.

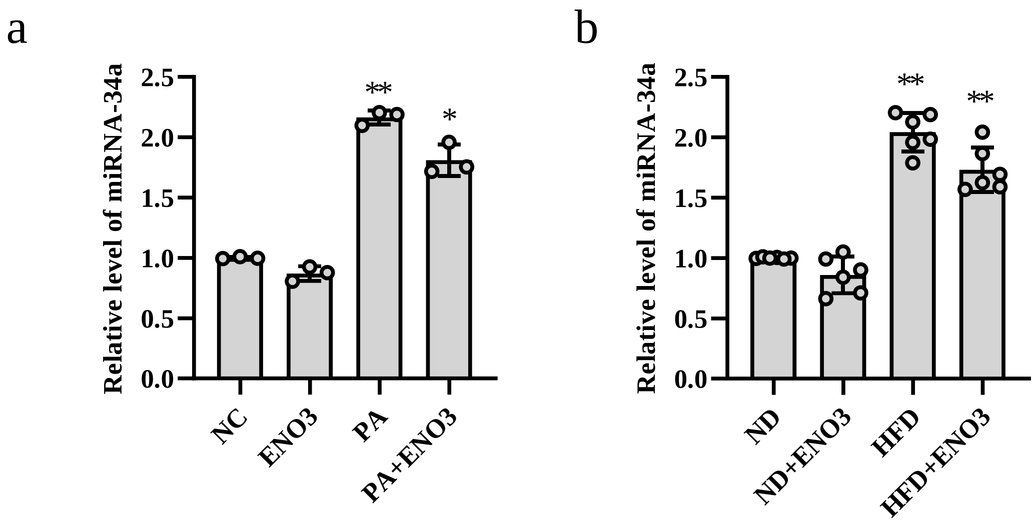

Supplementary Figure 8 The changes in the levels of miRNA-34a in AML-12 cells under different treatments (a) and in liver tissues of mice in each group (b). *P < 0.05 and **P < 0.01 vs NC/ND.

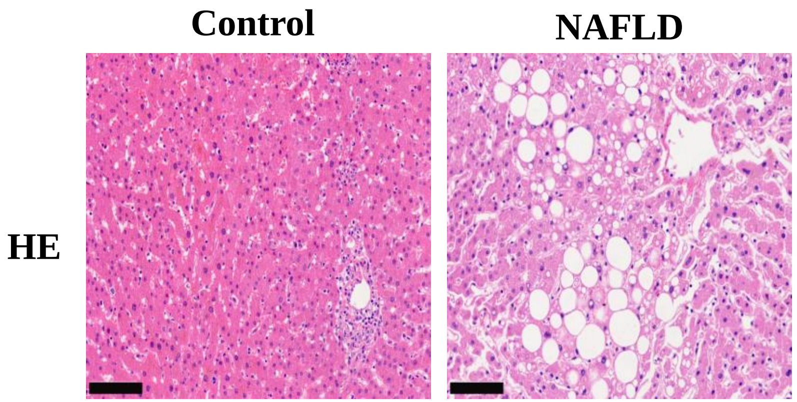

Supplementary Figure 9 HE staining of liver tissue (scale bars, 100 μM) in the liver of control subjects and patients with NAFLD.
