# Supplementary material for: MicroRNA-34a Mediates High-Fat-Induced Hepatic Insulin Resistance by Targeting ENO3
Source: Nutrients. 2023 Oct 31;15(21):4616. doi: 10.3390/nu15214616 (PMC10650923; doi:10.3390/nu15214616)
Supplement: Supplementary file 1 [file nutrients-15-04616-s001.zip › Supplementary Table.pdf]

MicroRNA-34a mediates high-fat-induced hepatic insulin resistance by targeting ENO3

Supplemental Table 1 The ingredients of normal diet and high-fat diet

Ingredients (g/kg)	Normal diet	High-fat diet
Corn Starch	495.692	290.692
Casein, 30Mesh	140	140
Maltodextrin 10	125	125
Sucrose	100	100
Soybean oil	40	40
Lard	0	205
Cellulose	50	50
Mineral mix (AIN-93M)	35	35
Vitamin mix (AIN-93M)	10	10
L-cystine	1.8	1.8
Choline bitartrate	2.5	2.5
t-Butylhydroquinone	0.008	0.008

Supplemental Table 2 The siRNA target sequences in this study

Name	Target Gene	siRNA target sequences (5’ to 3’)
siRNA-1	ENO3	GAGCTGTACAAGAACTTCA
siRNA-2	ENO3	GCAATTGCCTGCTCCTGAA
siRNA-3	ENO3	GAAACTAAGTGTTGTGGAT

Supplemental Table 3 Primer sequences used for quantitative real-time PCR

Gene/Primer	Sequences (5’ to 3’)
U6	
Reverse transcription	CGCTTCACGAATTTGCGTGTCAT
Forward	GCTTCGGCAGCACATATACTA
Reverse	CGCTTCACGAATTTGCGTGTC
miR-34a	
Reverse transcription	GTCGTATCCAGTGCGTGTCGTGGAGTCGGCAATTGCACTGGATACGACACAACC
Forward	GGGTGGCAGTGTCTTAGCT
Reverse	CAGTGCGTGTCGTGGAGT
β-actin	
Forward	GACGGCCAGGTCATCACTAT
Reverse	CGGATGTCAACGTCACACTT
ENO3	
Forward	ACAAAGCACGATACCTGGGG
Reverse	GCGATGTGTCGGTAGAGAGG

Supplemental Table 4 Basic information on mice treated with or without miR-34a or its inhibitor in each group.

Index	ND (n = 8)	ND + miR-34a (n = 8)	HFD (n = 8)	HFD + miR-34a inhibitor (n = 8)
Body weight (g)	36.845±2.432	38.096±2.962	46.826±3.151*	44.459±3.445*
TG (mmol/L)	0.405±0.126	0.413±0.126	0.610±0.133*	0.569±0.132*
TC (mmol/L)	3.459±0.772	3.403±0.867	4,986±0.932*	4.756±0.587*
HDL-C (mmol/L)	2.585±0.565	2.423±0.517	3.446±0.680*	3.398±0.416*
LDL-C (mmol/L)	0.530±0.209	0.550±0.264	1.141±0.313*	1.025±0.389*

TG, triglyceride; TC, total cholesterol; HDL-C, high-density lipoprotein cholesterol; LDL-C, low density lipoprotein cholesterol. *$P < 0.05$ vs ND.

Supplemental Table 5 Basic information on mice treated with or without overexpression of ENO3 in each group.

Index	ND (n = 10)	ND + ENO3 (n = 10)	HFD (n = 10)	HFD + ENO3 (n = 10)
Body weight (g)	34.344±1.880	33.176±2.106	46.497±2.021*	44.832±0.857*†
TG (mmol/L)	0.42±0.132	0.371±0.137	0.538±0.045*	0.417±0.078†
TC (mmol/L)	3.16±0.387	2.958±0.206	8.575±1.119*	7.833±1.157*
HDL-C (mmol/L)	2.605±0.309	2.495±0.180	5.328±0.355*	5.081±0.597*
LDL-C (mmol/L)	0.206±0.078	0.147±0.028	1.836±0.515*	1.640±0.342*

TG, triglyceride; TC, total cholesterol; HDL-C, high-density lipoprotein cholesterol; LDL-C, low density lipoprotein cholesterol. *, $P < 0.05$ vs ND, †$P < 0.05$ vs HFD.

Supplemental Table 6 Basic information of control subjects and NAFLD patients

Index	Control (n = 5)	NAFLD (n = 5)
Age (years)	50.00±10.95	49.00±3.81
Gender (male/female)	2/3	1/4
Systolic blood pressure (mmHg)	123.20±12.38	124.80±13.08
Diastolic blood pressure (mmHg)	82.60±10.31	82.40±8.35
BMI (kg/m^2)	22.07±1.61	26.56±3.96*
ALT (U/l)	14.40±3.58	31.20±15.56*

*, $P < 0.05$ vs Control.
